# Specificity effects in reasoning with counterintuitive and arbitrary conditionals

**DOI:** 10.3758/s13421-021-01235-5

**Published:** 2021-09-23

**Authors:** Lupita Estefania Gazzo Castañeda, Markus Knauff

**Affiliations:** Experimental Psychology and Cognitive Science, Justus Liebig University Giessen, Otto-Behaghel-Str. 10F, 35394 Giessen, Germany

**Keywords:** Conditional reasoning, Prior knowledge, Specificity

## Abstract

When people have prior knowledge about an inference, they accept conclusions from specific conditionals (e.g., “If Jack does sports, then Jack loses weight”) more strongly than for unspecific conditionals (e.g., “If a person does sports, then the person loses weight”). But can specific phrasings also elevate the acceptance of conclusions from unbelievable conditionals? In Experiment 1, we varied the specificity of *counterintuitive* conditionals, which described the opposite of what is expected according to everyday experiences (“If Lena/a person studies hard, then Lena/the person will not do well on the test”). In Experiment 2, we varied the specificity of *arbitrary* conditionals, which had no obvious link between antecedent and consequent (“If Mary/a person goes shopping, then Mary/ the person gets pimples”). All conditionals were embedded in MP and AC inferences. Participants were instructed to reason as in daily life and to evaluate the conclusions on a 7-point Likert scale. Our results showed a specificity effect in both experiments: participants gave higher acceptance ratings for specific than for unspecific conditionals.

Even subtle differences in phrasing can affect how people reason. Consider, for instance, the following two conditional inferences (cf. De Neys et al., 2002):

(1) If a person does sports, then the person loses weight.

A person does sports.

Therefore, the person loses weight.

(2) If Jack does sports, then Jack loses weight.

Jack does sports.

Therefore, Jack loses weight.

Both inferences have the same logical structure and also the same thematic content. From a logical point of view, they are actually equivalent. Yet there is a small but important difference in their phrasing: Inference (1) is phrased in an *unspecific* way (i.e., as a general rule without naming a specific person). Inference (2), instead, is phrased in a *specific* way and contains a specific person named Jack (see also Goodwin, 2014; Krzyżanowska et al., 2017; Quelhas et al., 2017).

At first sight, this small difference in the phrasing may appear irrelevant. Yet previous experiments have shown that the specificity of conditionals does indeed affect human reasoning: people judge specific conditionals as true more often than unspecific ones (Quelhas et al., 2017). And, more importantly, people also draw different conclusions from both kinds of conditionals. In previous experiments, we confronted participants with inferences of type (1) and (2) and encouraged them to reason as in daily life (Gazzo Castañeda & Knauff, 2019). That is, we did not ask participants to reason deductively, but to select the answer that they think most likely applies, according to their beliefs. The results were clear: participants accepted conclusions from specific conditionals more strongly than from unspecific conditionals. The conclusion in (2) was thus accepted more strongly than that in (1). We call this the *specificity effect*.

But what are the cognitive processes behind the specificity effect? In our previous work we have explained the specificity effect by an interplay between the pragmatics of language and the retrieval of prior knowledge from memory (Gazzo Castañeda & Knauff, 2019). We have argued that specific phrasings inhibit people’s use of prior knowledge, leading to overall higher acceptance ratings. However, this assumption has only been tested with *intuitive conditionals* (i.e., with contents that are familiar to the participants). But does the specificity effect also affect conditionals that we do *not* believe? Consider the following two inferences:

(3) If a person does sports, then the person becomes fat.

A person does sports.

Therefore, the person becomes fat.

(4) If Jack does sports, then Jack becomes fat.

Jack does sports.

Therefore, Jack becomes fat.

Again, the conditionals in (3) and (4) differ in their specificity. But now they describe something that disagrees with what we know from our daily life. It is, in fact, unlikely that somebody becomes fat when doing sports. Yet if the specificity effect also affects reasoning with unbelievable conditionals, then people should accept the conclusion in (4) more strongly than the one in (3). This, however, has not yet been tested empirically.

The goal of the present paper is to close this gap and to explore the specificity effect with unbelievable conditionals. Do specific phrasings also elevate the acceptance of conclusions that do not agree with our prior knowledge? Answering this question is important for two reasons. It will help to better understand the cognitive processes behind the specificity effect. And it gives more insight into the importance of prior knowledge in reasoning. This is important, as currently many researchers of the so-called new psychology of reasoning consider prior knowledge to be one of the main factors influencing the acceptability of inferences (e.g., Evans, 2012; Oaksford & Chater, 2020). Our idea, however, is twofold: if the phrasing of conditionals can elevate the acceptance of unbelievable conclusions, then this suggests (1) that human reasoning is not exclusively governed by prior knowledge, and (2) that subtle changes in the phrasing can reduce the impact of prior knowledge on reasoning. This would, if true, challenge reasoning theories based almost exclusively on prior knowledge.

The remainder of the paper is as follows: We begin with a brief overview of conditional reasoning and the role of prior knowledge in human reasoning. Then, we focus on the specificity of conditionals and describe how the pragmatics of language and prior knowledge interact when reasoning with conditionals. In the main part of the paper, we describe two experiments in which we varied the specificity of *counterintuitive* and *arbitrary* conditionals. Counterintuitive conditionals contained consequents that described the opposite of what is expected according to everyday knowledge (“If Lena/a person studies hard, then Lena/the person will not do well on the test”). Arbitrary conditionals had no obvious link between antecedents and consequents (“If Mary/a person goes shopping, then Mary/the person gets pimples”). For both experiments we predicted a specificity effect. Specific phrasings should decrease people’s readiness to consider their prior knowledge, resulting in higher acceptance ratings for specific than for unspecific conditionals, irrespective of their particular content.

## Prior knowledge in conditional reasoning

When people reason with conditionals in their daily life, they do not only consider the logical structure of the argument but also their own prior knowledge about the content. In inferences (1)–(4), for example, the conclusion follows from the logical rule of modus ponens (MP): if *p* then *q*; *p*; therefore *q*. But even though this inference is logically valid, people often use their prior knowledge about the content of such inferences, and know, for example, that although doing sports may lead to losing weight, this is not always the case (cf. Evans & Over, 2004; Johnson-Laird & Byrne, 2002; Oaksford & Chater, 2007). Hence, people sometimes refuse to accept that *q* (losing weight) necessarily follows from *p* (doing sports) (e.g., Byrne, 1989; Gazzo Castañeda & Knauff, 2018; Weidenfeld et al., 2005). Similarly, people also use their prior knowledge when evaluating logically invalid inferences such as:

(5) If a person does sports, then the person loses weight.

A person loses weight.

Therefore, the person does sports.

This inference is called affirmation of the consequent (AC: If *p* then *q*; *q*; therefore *p*). AC is invalid because according to classical logic a conditional only says that *q* follows from *p*, but not that *p* is necessary for *q*. Nevertheless, people often accept this logically invalid conclusion because they erroneously think that *p* does follow from *q*. However, when people consider their prior knowledge and realize that there are also other reasons besides doing sports (*p*) that can lead to weight loss (*q*), their acceptance of AC conclusions decreases (e.g., Daniel & Klaczynski, 2006; Quinn & Markovits, 1998; Rumain et al., 1983).

It is well known that these findings rely on the availability of *disabling* and *alternative conditions* (e.g., Cummins et al., 1991). Disabling conditions are circumstances that prevent *q* from happening although *p* is true. In our example, such disabling conditions are, for instance, bad nutrition, the wrong exercises, or metabolic issues. As shown by Thompson (1994, 1995, 2000), disabling conditions lower the perceived sufficiency of *p* for *q* and, as a consequence, lead to a lower acceptance of MP conclusions. Alternative conditions, by contrast, are circumstances besides *p* that also cause *q*. In our example, an alternative condition would be, for instance, going on a diet. Alternative conditions lower the perceived necessity of *p* for *q* and, as a consequence, lead to a lower acceptance of AC conclusions. Several studies have shown that the more disabling conditions a person knows, the less MP conclusions are accepted, and the more alternative conditions a person knows, the less AC conclusions are accepted (e.g., Cummins, 1995; Cummins et al., 1991; De Neys et al., 2002; De Neys et al., 2003b).

For a long time, people’s consideration of prior knowledge in reasoning was considered a bias and a source of error (e.g., Evans et al., 1983; Klauer et al., 2000; Wilkins, 1928). However, in the last years this has dramatically changed. Today, the use of prior knowledge is not considered a disruptive factor but rather an essential part of human reasoning (e.g., Evans, 2012; Oaksford, 2015; Oaksford & Chater, 2020). Consequently, many current researchers avoid deductive instructions that, in the past, often motivated people to pay more attention to the logical structure than to the content of the inference. Instead, most researchers now ask people to reason as in daily life, according to the criteria they personally find relevant (e.g., Bonnefond et al., 2014; Cummins, 1995; De Neys et al., 2002; De Neys et al., 2003b; Gazzo Castañeda & Knauff, 2018, 2019, 2020; Verschueren et al., 2005). The goal is to make experimental research more similar to *everyday reasoning.* For the same reason, participants’ responses are not evaluated as correct or false, but as acceptance ratings reflecting how strongly individuals believe in a conclusion according to their prior knowledge. In fact, such experiments have shown that people automatically consider prior knowledge during reasoning. They consider, not only the information in the premises, but also their world knowledge stored in long-term memory (Oaksford, 2015; see also Evans & Over, 2004; Vadeboncoeur & Markovits, 1999). For instance, they consider, not only the number of disabling and alternative conditions, but also how often such situations actually occur in daily life (Geiger & Oberauer, 2007; see also Fernbach & Erb, 2013). They might also take the strength of disablers or alternatives into account (e.g., that bad nutrition is probably a stronger disabler than metabolic issues; De Neys et al., 2003a; Quinn & Markovits, 1998).

## The specificity of conditionals

Disabling and alternative conditions are also important for the specificity effect: Both can be supported or inhibited by the more, or less, specific phrasing of the conditional. How this interaction might work, we already explained in Gazzo Castañeda and Knauff (2019). In our *two-phase approach* of conditional reasoning, the interaction proceeds in two steps: First, in the *pragmatic evaluation phase*, the pragmatics of language affect how a conditional is interpreted. Unspecific and specific conditionals have the same syntactic structure and thematic content. Therefore, people could, in principle, retrieve their prior knowledge about disabling and alternative conditions regarding the content of the conditional equally. However, people usually expect that information uttered to them has an informative value (Politzer, 1986; Sperber & Wilson, 1995; see also Grice, 1975). Therefore, people consider the exact wording of conditionals. When a conditional is unspecific, it refers to a person in general (i.e., “a person”). Accordingly, people interpret the conditional as a general rule that describes the overall relation between an antecedent and a consequent (e.g., sports and losing weight). However, when the conditional is specific and contains the name of a specific agent, people assume that there is a reason why this very specific agent is mentioned, and not another one. Specific conditionals are thus interpreted as rules about particular entities.

These different interpretations are carried over into the second phase, the *pragmatic application phase*. Here, the outcome of the pragmatic evaluation phase either encourages or inhibits reasoners to consider their prior knowledge about disabling and alternative conditions. In the case of unspecific conditionals, as these are interpreted as general rules, reasoners feel free to consider their general knowledge on disabling and alternative conditions. As a result, they lower their acceptance ratings. However, in the case of specific conditionals, their specific phrasing inhibits people’s consideration of prior knowledge. Reasoners think that there is a reason why this particular person is named, but, at the same time, they do not know this specific person. Therefore, they cannot know whether their prior knowledge on disabling and alternative conditions is helpful for this specific problem. Hence, they ultimately disregard their prior knowledge and accept the conclusion more strongly than for unspecific conditionals.

This explanation for the specificity effect means that the pragmatics of language inhibit the use of prior knowledge in the case of specific phrasings. But how far-reaching is the specificity effect? In Gazzo Castañeda and Knauff (2019), participants were confronted with conditionals whose content was intuitive: The conditionals always described relations that were known from everyday life, such in the examples (1) and (2). Therefore, the higher acceptance ratings for specific conditionals still agreed with participants’ prior knowledge: accepting more or less strongly that someone loses weight after doing sports is not in conflict with our experiences. But what happens when participants have to reason with conditionals that are contrary to everyday experiences? Let us return to example (4): “If Jack does sports, then Jack becomes fat.” This is certainly not what we expect from daily life. However, following our two-phase approach, we expect that people will also accept conclusions from such conditionals more strongly than without the specific phrasing. The reason is the following: Even though the conditional is unbelievable, the pragmatics of language should make people think that there is a reason why this particular person is mentioned. Maybe there are reasons why Jack, indeed, becomes fat when he does sports? Hence, people’s readiness to consider their prior knowledge should decrease with an increase of specificity. Therefore, we should obtain higher acceptance ratings for specific (“Jack”) than for unspecific (“a person”) conditionals, regardless of the believability of the inference.

We now present two experiments to test this prediction. In both experiments we confronted participants with either specific or unspecific conditionals and instructed them to reason as in daily life. Half of the problems were intuitive and agreed with prior knowledge. The other half of the problems were either counterintuitive (Experiment 1) or arbitrary (Experiment 2) and did not agree with prior knowledge. Counterintuitive conditionals described exactly the opposite of what could be expected according to prior knowledge (e.g., “If a person [Jenni] goes on a diet, then the person [Jenni] does not lose weight”). Arbitrary conditionals described relations without any obvious relation between antecedent and consequent (e.g., “If a person [Mary] goes shopping, then the person [Mary] gets pimples”). We expected people to accept conclusions from specific conditionals more strongly than conclusions from unspecific conditionals, irrespective of their content.

## Experiment 1

### Method

#### Participants

All participants were recruited online via Prolific (www.prolific.co). In total, 100 participants took part, but 13 participants had to be excluded from the final sample because they did not meet the inclusion criteria: being native German speakers, having no prior knowledge of formal logic, and reporting to have worked focused and conscientiously during the experiment. The final sample thus consisted of 87 participants (34 = female, 52 = male, one = other), with a mean age of 26.98 years (*SD* = 6.49). Forty-one of the participants were confronted only with unspecific problems, and 46 only with specific problems. Which problems were assigned to which participants was determined randomly. Overall, 75 participants indicated a higher education entrance qualification as their academic level, and 12 participants indicated a secondary school certificate.

#### Material and design

We took eight conditionals from the literature (from Cummins, 1995; De Neys et al., 2002; Verschueren et al., 2005) and phrased them either with their original consequent (e.g., “If a person goes on a diet, then the person loses weight”), or with the negated consequent (e.g., “If a person goes on a diet, then the person does not lose weight”). Thus, while the conditionals with the original consequent were intuitive and agreed with prior knowledge, the ones with negated consequents were counterintuitive and did not agree with prior knowledge. Each conditional was embedded in an MP and an AC inference and presented to participants either with an unspecific or a specific agent. Unspecific agents had no concrete name but referred to a general person (i.e., “a person”). Specific agents were always persons with a specific name (e.g., Jack, Anne, . . .). We took care to always use different names for all our specific conditionals. Overall, each participant worked on 32 problems. An overview of our conditionals can be found in Table 1. All problems were presented in German.

**Table 1:** Conditionals used in Experiment 1

Intuitive conditionals Unspecific If a person drinks much cola, then the person gets fat. If a person sits in the draught, then the person catches a cold. If a person reads without glasses, then the person gets a headache. If a person brushes his teeth, then the person does not get cavities. If a person turns on the air conditioner, then the person feels cool. If a person goes on a diet, then the person loses weight. If a person studies hard, then the person will do well on the test. If a person drinks coffee in the evening, then the person will have difficulties falling asleep.	
Specific If Bruno drinks much cola, then Bruno gets fat. If Emma sits in the draught, then Emma catches a cold. If Daniel reads without glasses, then Daniel gets a headache. If Julia brushes her teeth, then Julia does not get cavities. If Stefan turns on the air conditioner, then Stefan feels cool. If Laura goes on a diet, then Laura loses weight. If Sarah studies hard, then Sarah will do well on the test. If Alex drinks coffee in the evening, then Alex will have difficulties falling asleep.	
Counterintuitive conditionals Unspecific If a person drinks much cola, then the person does not get fat. If a person sits in the draught, then the person does not catch a cold. If a person reads without glasses, then the person does not get a headache. If a person brushes his teeth, then the person gets cavities. If a person turns on the air conditioner, then the person does not feel cool. If a person goes on a diet, then the person does not lose weight. If a person studies hard, then the person will not do well on the test. If a person drinks coffee in the evening, then the person will not have difficulties falling asleep.	
Specific If Thomas drinks much cola, then Thomas does not get fat. If Claudia sits in the draught, then Claudia does not catch a cool. If Florian reads without glasses, then Florian does not get a headache. If Anna brushes her teeth, then Anna gets cavities. If Philipp turns on the air conditioner, then Philipp does not feel cool. If Jenni goes on a diet, then Jenni does not lose weight. If Lena studies hard, then Lena will not do well on the test. If Kai drinks coffee in the evening, then Kai will not have difficulties falling asleep.	

*Note.* All conditionals had the form “If *p*, then *q*” and were embedded in the inferences modus ponens (MP: “If *p* then *q*; *p*; *q*”) and affirmation of the consequent (AC: “If *p* then *q*; *q*; *p*”). For example: “If Laura goes on a diet, then Laura loses weight. Laura goes on a diet. Laura loses weight” (for MP) and “If Laura goes on a diet, then Laura loses weight. Laura loses weight. Laura goes on a diet” (for AC). The original materials were in German language. The conditionals were adapted from Cummins (1995), De Neys et al. (2002), and Verschueren et al. (2005)

The experiment followed a 2 (specificity: unspecific vs. specific) × 2 (content: intuitive vs. counterintuitive) × 2 (inference: MP vs. AC) mixed design. The specificity was varied between subjects, all other variables within subjects. As the dependent variable, we asked the participants to indicate how certain they are that the conclusion can be drawn. They could indicate their answer on a 7-point Likert scale (1 = *very uncertain* to 7 = *very certain*). For example:

If a person goes on a diet, then the person loses weight.

A person goes on a diet.

The person loses weight.

How certain are you that this conclusion can be drawn?

Very uncertain 1 – 2 – 3 – 4 – 5 – 6 – 7 Very certain

#### Procedure

The experiment was conducted online via SoSci Survey (www.soscisurvey.de; Leiner, 2014). The problems were presented at once on the screen together with the response scale. The premises were presented in black and the conclusion in red. Importantly, as in Gazzo Castañeda and Knauff (2019), participants were told that there are no right or wrong answers. They should answer as they would in daily life and select the answer that they think is most likely to apply. This information was highlighted in boldface. We also told participants that it is possible that they may get the feeling that some problems are very similar or showed repeatedly, but that they should nevertheless try to consider each problem separately and independently from the ones presented previously. In addition, we also warned them that some problems can contain negations, so they should read carefully. The 32 inference problems were presented randomly. At the beginning of the experiment, we presented a practice problem consisting of one MP inference with an unspecific or specific term, respectively.

### Results

**Fig. 1:**
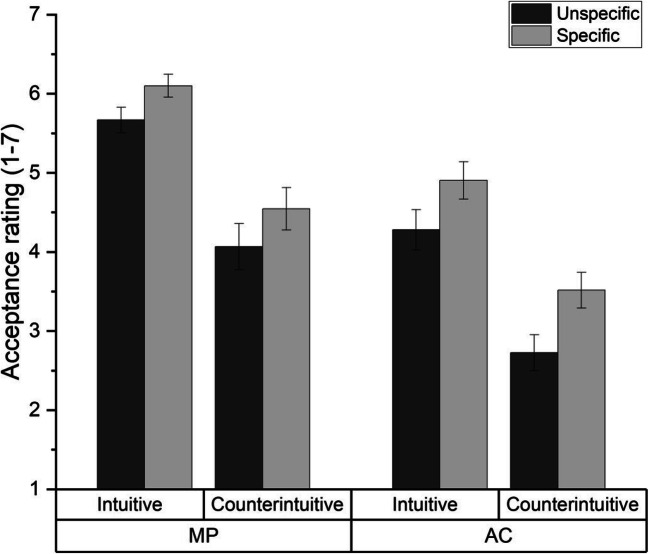
Acceptance ratings (1–7) for MP and AC inferences for specific and unspecific, intuitive, and counterintuitive conditionals, in Experiment 1. Error bars show standard errors

We analyzed participants’ acceptance ratings with a 2 (specificity: unspecific vs. specific) × 2 (content: intuitive vs. counterintuitive) × 2 (inference: MP vs. AC) mixed analysis of variance (ANOVA). Descriptive statistics can be found in Fig. 1.

The ANOVA revealed a main effect of specificity, *F*(1, 85) = 6.15, *p* = .015, η_p_^2^ = .067, a main effect of content, *F*(1, 85) = 126.07, *p* < .001, η_p_^2^ = .597, and a main effect of inference, *F*(1, 85) = 50.94, *p* < .001, η_p_^2^ = .375. As expected, specific conditionals (*M* = 4.77, *SD* = 1.12) were accepted more strongly than unspecific conditionals (*M* = 4.19, *SD* = 1.06). Furthermore, intuitive conditionals (*M* = 5.25, *SD* = 1.09) were accepted more strongly than counterintuitive conditionals (*M* = 3.73, *SD* = 1.46), and MP inferences (*M* = 5.11, *SD* = 1.32) were accepted more strongly than AC inferences (*M* = 3.88; *SD* = 1.45). The ANOVA revealed no interactions between content and specificity: *F*(1, 85) = 0.15, *p* = .697, η_p_^2^ = .002; between inference and specificity: *F*(1, 85) = 0.53, *p* = .471, η_p_^2^ = .006; between content and inference: *F*(1, 85) = 0.98, *p* = .325, η_p_^2^ = .011; or between content, specificity, and inference: *F*(1, 85) = 0.32, *p* = .575, η_p_^2^ = .004.

### Discussion

We found a main effect of specificity: In both intuitive *and* counterintuitive conditionals, participants accepted conclusions from specific conditionals more strongly than from unspecific conditionals. Remember that participants were instructed to reason as in daily life. So, they could have used their prior knowledge and given only low acceptance ratings, in particular when reasoning with counterintuitive conditionals. That they did not do so when these were phrased in a specific way is somehow surprising, given the robustness of content effects in the psychology of reasoning (for overviews, see Ball & Thompson, 2018; Evans et al., 1993; Klauer et al., 2000). Markovits and Vachon (1989), for instance, conducted experiments with so-called contrary-to-fact conditionals, which describe the opposite of what one would expect from everyday experiences (e.g., “If one hits a glass with a feather, then the glass will break”). They found that prior knowledge made reasoning more difficult (see also Dias & Harris, 1988, 1990; Markovits, 2014; Markovits & Lortie-Forgues, 2011). In fact, our counterintuitive conditionals are similar to Markovits’ and Vachon’s contrary-to-fact conditionals, as our problems also described the opposite of what one could expect from everyday experience. Yet, in our experiment we varied the specificity of these conditionals and showed that even for such conditionals specific phrasings led to higher acceptance ratings. In other words, the rather robust effect of prior knowledge on reasoning can be suppressed by the specificity of the conditional.

One possible problem with our counterintuitive conditionals is that they were created by negating the consequents of conditionals. This does indeed lead to conditionals that are the opposite of what we usually expect, which was our intention. Yet this does not mean that the negated conditionals are always the exact opposite of our prior knowledge. Although it is weird that someone gets fat when doing sports, this is still possible (e.g., if the person always eats fries after training). Another problem might be that by negating the consequent of intuitive conditionals we somehow also changed the logical structure of the conditionals. While intuitive conditionals had the logical form “If *p* then *q*,” counterintuitive conditionals could be understood as “If *p* then not *q.*” This might not be problematic for our research, as we concentrated on the specificity effect *within* intuitive or counterintuitive conditionals and did not make comparisons between both types of conditionals. Nevertheless, we wanted to control this in the next experiment by using inferences that (1) had definitely no relation to our prior knowledge and (2) did not contain negations.

## Experiment 2

Instead of counterintuitive conditionals, we now used conditionals to which we refer as *arbitrary conditionals*. Our idea was that these conditionals avoid any connection to prior knowledge because there is no obvious link between the antecedent and the consequent. Some researchers call these conditionals missing-link conditionals (see, e.g., Skovgaard-Olsen et al., 2016, 2017). The arbitrary conditionals we used in this experiment always described an action of an agent in the antecedent together with an unrelated event in the consequent (e.g., “If a person eats too much rice, then the person sings a song”). These conditionals were thus not only arbitrary but also described something that one would not expect in daily life. Therefore, when participants are confronted with unspecific arbitrary conditionals and are instructed to reason as in daily life, they should assign very low acceptance ratings. However, if people do indeed suppress their prior knowledge when reasoning with specific conditionals, then introducing a specific agent should increase acceptance ratings.

### Methods

#### Participants

Participants were recruited online via Prolific (www.prolific.co) and had English as a first language. One hundred and twenty participants took part, but 11 participants had to be excluded from the final sample because they did not meet the inclusion criteria: having no prior knowledge of formal logic and reporting to have worked focused and conscientiously during the experiment. The final sample thus consisted of 109 participants (78 = female, 30 = male, one = other) with a mean age of 28.04 years (*SD* = 6.38). Fifty-three participants were confronted only with unspecific problems, 56 only with specific ones. Which problems were assigned to which participants was determined randomly. Overall, 32 participants indicated having a high school degree, 76 participants indicated having an academic degree or higher, and one participant did not specify.

#### Materials and design

Our experiment consisted of 16 conditionals. Half were in accordance to prior knowledge and had the same content as the intuitive conditionals from Experiment 1. The other half were arbitrary conditionals that described conditional relations that do not have any correspondence to everyday life (e.g., “If a person goes shopping, then the person gets pimples”). Each conditional was embedded in an MP and an AC inference and presented to participants either with an unspecific or a specific agent. Overall, participants thus solved 32 problems. In contrast to Experiment 1, now all problems were presented in the English language. An overview of our conditionals can be found in Table 2.

**Table 2:** Conditionals used in Experiment 2

Intuitive conditionals Unspecific If a person drinks too much soda, then the person gets fat. If a person sits in the draught, then the person catches a cold. If a person reads without glasses, then the person gets a headache. If a person brushes their teeth, then the person avoids cavities. If a person turns on the air conditioner, then the person feels cool. If a person goes on a diet, then the person loses weight. If a person studies hard, then the person does well on the test. If a person drinks coffee in the evening, then the person has difficulties falling asleep.	
Specific If Peter drinks too much soda, then Peter gets fat. If Zoe sits in the draught, then Zoe catches a cold. If Sophie reads without glasses, then Sophie gets a headache. If James brushes his teeth, then James avoids cavities. If Will turns on the air conditioner, then Will feels cool. If Tara goes on a diet, then Tara loses weight. If Jane studies hard, then Jane does well on the test. If Harry drinks coffee in the evening, then Harry has difficulties falling asleep.	
Arbitrary conditionals Unspecific If a person takes a bus, then the person wears a blue pullover. If a person feels tired, then the person starts to laugh. If a person eats too much rice, then the person sings a song. If a person goes shopping, then the person gets pimples. If a person gets an invitation, then the person feels thirsty. If a person buys a book, then the person gains weight. If a person goes to a pub, then the person gets rich. If a person eats pizza, then the person takes a shower.	
Specific If Anne takes a bus, then Anne wears a blue pullover. If John feels tired, then John starts to laugh. If Fred eats too much rice, then Fred sings a song. If Mary goes shopping, then Mary gets pimples. If Jenny gets an invitation, then Jenny feels thirsty. If Jake buys a book, then Jake gains weight. If Jack goes to a pub, then Jack gets rich. If Linda eats pizza, then Linda takes a shower.	

*Note.* All conditionals had the form “If *p*, then *q*” and were embedded in the inferences modus ponens (MP: “If *p* then *q*; *p*; *q*”) and affirmation of the consequent (AC: “If *p* then *q*; *q*; *p*”). For example: “If Tara goes on a diet, then Tara loses weight. Tara goes on a diet. Tara loses weight” (for MP) and “If Tara goes on a diet, then Tara loses weight. Tara loses weight. Tara goes on a diet” (for AC). The intuitive conditionals were adapted from Cummins (1995), De Neys et al. (2002), and Verschueren et al. (2005)

The experiment followed a 2 (specificity: unspecific vs. specific) × 2 (content: intuitive vs. arbitrary) × 2 (inference: MP vs. AC) mixed design. The specificity was varied between participants, all other variables within participants. Participants had to indicate how certain they are that they can draw the conclusion. As in Experiment 1, they could indicate their answer on a 7-point Likert scale (1 = *very uncertain* to 7 = *very certain*).

#### Procedure

The procedure was the same as in Experiment 1. The experiment was conducted online via SoSci Survey (Leiner, 2014), and participants were instructed to evaluate the conclusion as in everyday life and to pick the answer that applied best according to their opinion.

### Results

**Fig. 2:**
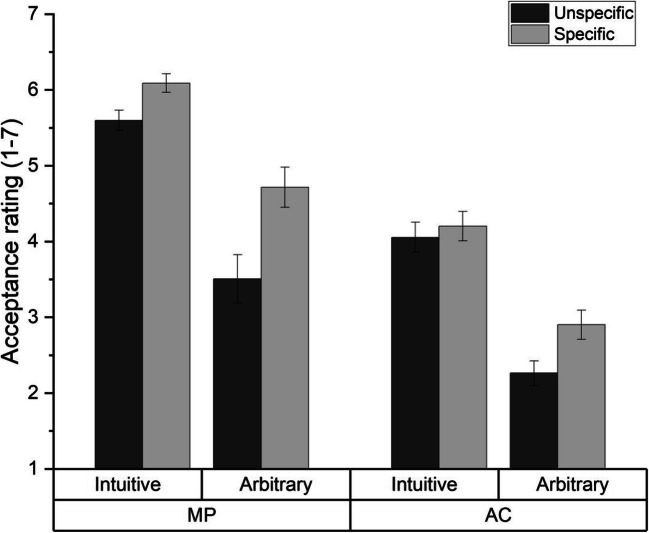
Acceptance ratings (1–7) for MP and AC inferences for specific and unspecific, intuitive, and arbitrary conditionals, in Experiment 2. Error bars show standard errors

We analyzed participants’ acceptance ratings with a 2 (specificity: unspecific vs. specific) × 2 (content: intuitive vs. arbitrary) × 2 (inference: MP vs. AC) mixed ANOVA. Descriptive statistics can be found in Fig. 2.

The ANOVA revealed a main effect of specificity, *F*(1, 107) = 12.59, *p* = .001, η_p_^2^ = .105, a main effect of inference, *F*(1, 107) = 81.64, *p* < .001, η_p_^2^ = .433, and a main effect of content, *F*(1, 107) = 127.23, *p* < .001, η_p_^2^ = .543. Again, specific conditionals (*M* = 4.48, *SD* = 0.93) were accepted more strongly than unspecific conditionals (*M* = 3.86, *SD* = 0.90). MP inferences (*M* = 4.99, *SD* = 1.49) were accepted more strongly than AC inferences (*M* = 3.36, *SD* = 1.18). And intuitive conditionals (*M* = 4.99, *SD* = 0.84) were accepted more strongly than arbitrary conditionals (*M* = 3.36, *SD* = 1.53). This time, however, we also found an interaction between specificity and content, *F*(1, 107) = 4.30, *p* = .041, η_p_^2^ = .039. As can be seen in Fig. 2, the specificity effect was more pronounced for arbitrary conditionals (*M*_unspecific_ = 2.89, *SD*_unspecific_ = 1.47; *M*_specific_ = 3.81, *SD*_specific_ = 1.46), *t*(107) = 3.29, *p* = .001, *d* = 0.631,^1^ than for intuitive conditionals (*M*_unspecific_ = 4.83, *SD*_unspecific_ = 0.80; *M*_specific_ = 5.15, *SD*_specific_ = 0.85), *t*(107) = 2.02, *p* = .046, *d* = 0.387 (Bonferroni-adjusted alpha: 0.025). We also found an interaction between content and inference, *F*(1, 107) = 4.58, *p* = .035; η_p_^2^ = .041: people’s higher acceptance for MP inferences compared to AC inferences was slightly more pronounced for intuitive (*M*_MP_ = 5.85, *SD*_MP_ = 0.97; *M*_AC_ = 4.13, *SD*_AC_ = 1.45) than for arbitrary conditionals (*M*_MP_ = 4.13, *SD*_MP_ = 2.23; *M*_AC_ = 2.59, *SD*_AC_ = 1.34), although both differences were significant, *t*(108) = 9.94, *p* < .001, *d* = 1.40; *t*(108) = 7.81, *p* < .001, *d* = 0.80, respectively (Bonferroni-adjusted alpha: 0.025). The ANOVA revealed no interactions between inference and specificity: *F*(1, 107) = 1.61, *p* = .207, η_p_^2^ = .015; or between specificity, inference, and content: *F*(1, 107) = 1.70, *p* = .196, η_p_^2^ = .016.

### Discussion

Our results agree nicely with those of Experiment 1. Again, participants gave higher acceptance ratings to conclusions from specific than from unspecific conditionals. Moreover, the specificity effect was even more pronounced for arbitrary than for intuitive conditionals. This corroborates our two-phase approach: The content of arbitrary conditionals had no correspondence with our daily life experience, which led to very low acceptance ratings for unspecific arbitrary conditionals. However, when these conditionals were phrased with a specific agent, people disregarded their prior knowledge so that the arbitrary content of the conditionals lost relevance. As a result, participants gave much higher acceptance ratings than for unspecific arbitrary conditionals, resulting in a pronounced specificity effect. For intuitive conditionals, however, this is different. Although specific phrasings also made participants ignore their prior knowledge, the specificity effect was less pronounced under this condition because these conditionals did not conflict with prior knowledge. Intuitive conditionals always agree with everyday-life experience, even when disabling and alternative conditions are considered.

Another observation is that participants accepted MP inferences more strongly than AC inferences (which was also the case in Experiment 1). As participants were not instructed to reason logically, this might appear surprising at first sight. However, such directionality effects in reasoning are well known (e.g., Espino et al., 2015; Grosset & Barrouillet, 2003; Oberauer et al., 2005). Even if our participants were instructed to reason as in daily life, a conditional of the form “if *p* then *q*” suggests that the next premise should contain *p*, thus enhancing people’s acceptance of MP inferences (see Bonnefond et al., 2012; Bonnefond & Van der Henst, 2009). Moreover, it could also have been that alternatives were more easily available than disabling conditions, thus resulting in an overall lower acceptance of AC inferences. When we selected the content of our intuitive conditionals, we took care that they had a comparable number of disabling and alternative conditions, but we did not control for this statistically. Therefore, it might be that it was easier for participants to think of reasons why *q* might be the case without *p* than otherwise. Nevertheless, it is also worth mentioning that under some conditions, AC inferences were accepted to a similar extent as MP inferences (at least descriptively)—namely, when the content of the AC inference was intuitive (and specific) and the content of the MP inference was arbitrary (and unspecific). This shows once more the importance of specificity and prior knowledge in reasoning.

One unexpected finding from Experiment 2 is, however, that for AC inferences with intuitive conditionals, the specificity effect was not as pronounced as in Experiment 1. This is surprising since we used the same intuitive conditionals for both experiments. In fact, most of our intuitive conditionals were also used in Gazzo Castañeda and Knauff (2019), where we were able to find specificity effects in three independent experiments. We are thus not sure why this time we were not able to find a specificity effect for AC inferences with intuitive content. Possible reasons are slight differences in the phrasing resulting from the translation of our materials to the English language, or differences in the samples (German vs. English native speakers). We will explore this in future studies.

## General discussion

We conducted two experiments to explore the generality of the specificity effect*.* The conditionals we used in our experiments had content that either agreed or disagreed with people’s prior knowledge. Our main finding is that the specificity effect is not limited to conditionals for which people have prior knowledge. It is clearly visible even when people reason with counterintuitive and arbitrary conditionals. In the former case, the conditional said something that was exactly the opposite of what we know from daily life. In the latter case, there was no obvious link between the antecedent and the consequent that would allow people to use their prior knowledge. These findings have important implications.

The first implication of our results is that, as is well known, people do not always follow the norms of classical logic. People do not only consider the logical structure of an inference but also the content of conditionals. This again demonstrates the nonmonotonic and defeasible nature of human reasoning (e.g., Gazzo Castañeda & Knauff, 2021; Johnson-Laird & Ragni, 2019; Oaksford & Chater, 2013; Pollock, 1987; Stenning & van Lambalgen, 2005). The results also agree with the main assumptions of the new psychology of reasoning (Evans, 2012; Oaksford & Chater, 2020; Over, 2009). But they do so only partially. Yes, our participants were instructed to reason as in daily life, and they used their prior knowledge to assign degrees of belief to the content of an inference. Obviously, this affected how strongly they accepted the conclusions. But, no, they did not do so all the time, as the new psychology of reason assumes. Rather, participants’ prior knowledge could be suppressed by the phrasing of conditionals. Apparently, people consider the way a conditional is phrased and assume that there is always a reason why a conditional is phrased in a particular way and not in another. This is important because previous research mainly focused on how the instructions (e.g., George, 1995; Stevenson & Over, 1995; Vadeboncoeur & Markovits, 1999) and the response modalities (Markovits et al., 2010) influence the effect of prior knowledge in reasoning. Some even explored how emotions affect the consideration of potential disabling conditions (Gazzo Castañeda et al., 2016; Gazzo Castañeda & Knauff, 2016). To the best of our knowledge, however, this is the only work showing that subtle differences in phrasings can limit the effect of prior knowledge and elevate the acceptance of unbelievable conditionals.

The second implication of our results is that they help us to understand the particular cognitive processes that lie behind the specificity effect. In Gazzo Castañeda and Knauff (2019) we argued that specific phrasings inhibit people’s consideration of their prior knowledge of disabling and alternative conditions, resulting in higher acceptance ratings. Now, we also found specificity effects for counterintuitive and arbitrary conditionals. This is important because for such conditionals we actually do not know any particular disabling and alternative conditions. Counterintuitive conditionals, for instance, describe the opposite of what we know from everyday experience (e.g., “If a person does sports, then the person does not lose weight”). It is thus difficult to think of particular reasons of why *p* would not lead to *q* (or *q* would not be caused by *p*), because the described relationship is already unbelievable. This becomes even more relevant for arbitrary conditionals. Arbitrary conditionals describe relations where there is no obvious link between *p* and *q* (e.g., “If a person eats pizza, then the person takes a shower”). Therefore, reasoners should not be able to think of particular disabling or alternative conditions as they do not have prior knowledge on the particular conditional relationship. Nevertheless, participants did accept conclusions from such conditionals more strongly when they were phrased in a specific way. This suggests that the specificity effect does not merely inhibit people’s consideration of disabling and alternative conditions, but inhibits people’s general attempt to evaluate conditionals with regard to their prior knowledge. In light of specific phrasings, people refrain from questioning the content of inferences, which results in higher acceptance ratings compared to unspecific phrasings. Moreover, in case of counterintuitive or arbitrary conditionals, it may even be that participants not only do not question the content of the inferences but also try to seek explanations for their counterintuitive or arbitrary contents (e.g., Johnson-Laird et al., 2004; Khemlani & Johnson-Laird, 2012).

The third implication of our study is that the specificity effect is more far-reaching than one might think. Of course, we are not the first who emphasize the differences between specific and unspecific conditionals (e.g., Gazzo Castañeda & Knauff, 2019; Goodwin, 2014; Krzyżanowska et al., 2017; Quelhas et al., 2017). Most research on the phrasing of conditionals, however, was concerned with the meaning of conditionals rather than with reasoning. Quelhas et al. (2017), for instance, showed that specific conditionals are more often judged a priori as true as unspecific ones. Similarly, Goodwin (2014) investigated the interpretation of conditionals, but found no differences between specific and unspecific conditionals. More important for our findings, however, is the idea that specific and unspecific conditionals differ in their scope. Evans et al. (2003), and later also Goodwin (2014), argued that unspecific conditionals are wide-scope conditionals and can thus be falsified more easily: the presence of any *p-*and-not-*q* case is enough to render the conditional false. Specific conditionals, in contrast, are narrow-scope conditionals and are more difficult to falsify: Specific conditionals are not falsified by the mere possibility that a *p*-and-not-*q* case *might* exist, but rather only falsified if such a *p*-and-not-*q* case actually arises. In principle, the idea that specific and unspecific conditionals differ in how easy they can be falsified is compatible with our findings. The higher acceptance ratings for specific conditionals suggest that these were indeed more difficult to falsify than unspecific conditionals. However, our findings go beyond Evans et al. (2003) and Goodwin’s (2014) proposal. While they only discussed theoretically why specific and unspecific conditionals should differ in terms of how easily they can be falsified, we now offer experimental support that people do indeed falsify unspecific conditionals more easily than specific ones (as these conditionals receive lower acceptance ratings).

The most general corollary from our study is: Be careful when designing your reasoning materials. Ignoring the potential effects of the phrasing of conditionals may lead to biased results and thus to incorrect cognitive theories of human reasoning (Gazzo Castañeda & Knauff, 2019). Particularly important for future research is the specificity effect we found for arbitrary conditionals. In current research, arbitrary conditionals are often used to avoid effects of prior knowledge on reasoning. By not describing any meaningful relationship between antecedent and consequent, researchers aim to capture people’s pure reasoning competence (e.g., Evans et al., 1993; Markovits et al., 2019; Noveck et al., 2004). Accordingly, such conditionals often have been used to investigate developmental trends in reasoning (Barrouillet & Lecas, 1998, 1999; Markovits & Barrouillet, 2002; but see Markovits et al., 2016). We do not want to deny here that most arbitrary conditionals may indeed be less prone to content effects. But we should also be aware that even such conditionals can be affected by the specificity effect, as the present research shows. Our findings are just one example, but there are certainly many other supposedly small factors that can contaminate the outcome of your reasoning experiments.

## Footnotes

1 Standardized mean differences (*d*) were computed as described by Borenstein (2009).
